# Development of an Apple Snack Enriched with Probiotic *Lacticaseibacillus rhamnosus*: Evaluation of the Refractance Window Drying Process on Cell Viability

**DOI:** 10.3390/foods13111756

**Published:** 2024-06-03

**Authors:** Helena Nuñez, Aldonza Jaques, Karyn Belmonte, Jamil Elitin, Mónika Valdenegro, Cristian Ramírez, Andrés Córdova

**Affiliations:** 1 Departamento de Ingeniería Química y Ambiental, Universidad Técnica Federico Santa María, P.O. Box 110-V, Valparaíso 2390123, Chile; helena.nunez@usm.cl (H.N.); aldonza.jaques@usm.cl (A.J.); karyn.belmonte.12@sansano.usm.cl (K.B.); jamil.elitin.13@sansano.usm.cl (J.E.); cristian.ramirez@usm.cl (C.R.); 2 Programa de Doctorado de Ciencias Agroalimentarias, Facultad de Ciencias Agronómicas y de los Alimentos, Pontifica Universidad Católica de Valparaíso, Valparaíso 2340025, Chile; monika.valdenegro@pucv.cl; 3 Escuela de Agronomía, Pontificia Universidad Católica de Valparaíso, Calle San Francisco S/N, La Palma, Quillota 2260000, Chile; 4 Escuela de Alimentos, Pontificia Universidad Católica de Valparaíso, Waddington 716 Playa Ancha, Valparaíso 2340025, Chile

**Keywords:** probiotics *Lacticaseibacillus rhamnosus*, Refractance Window (RW**^TM^**), dried apples, vacuum impregnation

## Abstract

The objective of this study was to develop a dried apple snack enriched with probiotics, evaluate its viability using Refractance Window (RW^TM^) drying, and compare it with conventional hot air drying (CD) and freeze-drying (FD). Apple slices were impregnated with *Lacticaseibacillus rhamnosus* and dried at 45 °C using RW^TM^ and CD and FD. Total polyphenol content (TPC), color (∆E*), texture, and viable cell count were measured, and samples were stored for 28 days at 4 °C. Vacuum impregnation allowed for a probiotic inoculation of 8.53 log CFU/gdb. Retention values of 6.30, 6.67, and 7.20 log CFU/gdb were observed for CD, RW^TM^, and FD, respectively; the population in CD, RW^TM^ remained while FD showed a decrease of one order of magnitude during storage. Comparing RW^TM^ with FD, ∆E* was not significantly different (*p* < 0.05) and RWTM presented lower hardness values and higher crispness than FD, but the RW^TM^-dried apple slices had the highest TPC retention (41.3%). Microstructural analysis showed that RW^TM^ produced a smoother surface, facilitating uniform moisture diffusion and lower mass transfer resistance. The effective moisture diffusion coefficient was higher in RW^TM^ than in CD, resulting in shorter drying times. As a consequence, RW^TM^ produced dried apple snacks enriched with probiotics, with color and TPC retention comparable to FD.

## 1. Introduction

Probiotics are defined as living microorganisms that, when ingested in sufficient quantities, exert a positive effect on health and wellbeing, including balancing the intestinal flora and enhancing the immune system [1,2]. The most commonly used microorganisms with probiotic characteristics are lactic acid bacteria (LAB), particularly *Lacticaseibacillus* species such as *L. casei*, *L. plantarum*, *L. rhamnosus,* and *L. acidophilus* [3,4,5]. *Bifidobacterium* species (*B. longum*, *B. animali,* and *B. lactis*) are the other most known probiotic microorganisms [3]. The most commonly used carrier matrices are dairy products, probably because of their acidity and pH, which are the most suitable parameters for probiotics [6]. However, an increasing interest in nondairy probiotic products can be attributed to several factors reflecting both changing consumer preferences and broader industry trends. Some of the key reasons for this are lactose intolerance, allergies, cholesterol content, cultural beliefs (strict vegan), and specific religious beliefs among certain communities [6,7,8,9]. On the other hand, using nondairy food matrices as probiotic carriers can indeed offer several advantages, such as the potential for these matrices to provide a protective environment for probiotic bacteria [10]. This protection can reduce the exposure of these plants to harsh gastrointestinal conditions, enhancing their viability and effectiveness [9]. In this respect, fruits and vegetables have been studied as interesting alternative vehicles for probiotic use since they already contain abundant bioactive compounds; thus, their use can be enhanced by the incorporation of probiotics, resulting in the development of nutritious foods with dual health-promoting benefits [8,11,12,13].

Vacuum impregnation (VI) can be used for the production of functional foods through the impregnation of a liquid that is rich in bioactive compounds and specific minerals that aid in nutritional quality [11]. The porous structure of fruits and vegetables, with their intercellular spaces, allows them to be effectively impregnated with external solutions. This process is valuable for developing high-quality food products with desired attributes and characteristics [14,15]. Several investigations have used this technology for the incorporation of probiotics in fruits, for example, in apples [16,17,18,19,20], murta [21], melon [22], bananas [23], apricots [24], and Andean blackberries [25]. Additionally, drying offers several advantages for preserving enriched fruit and vegetable snacks, including cost savings in storage, packaging, and transportation, and extended shelf life [26,27]. In this regard, the Refractance Window (RW^TM^) is a fourth-generation drying technology that has gained much attention in recent years due to its several advantages [28,29]. The products, either in the form of pulp, puree, paste, or sliced, are spread over a flexible polyester film (e.g., Mylar), which is placed above hot water, and the drying process occurs by means of conduction and radiation [30]. This is an efficient drying technology with a higher drying rate and shorter drying time than other drying methods. Compared with hot air drying, this new technology has a positive impact on the retention of bioactive compounds [28], as demonstrated by the drying of apples [30,31], carrots [32], tomato paste [33], cherry pulp [34], mangoes [35], blueberries [26], and kiwis [36], resulting in products obtained with greater retention of nutrients and reduced drying time. However, it is worth pointing out some disadvantages of RW^TM^, such as low production capacity and requiring the cleaning of the belt before or after a new process. It is also not recommended for materials with high sugar content due to the adhesive behavior of the pulp. Due to the low glass transition temperature of the material, it is important to choose the appropriate material to dry and select the film: the options are Mylar film or another material with a low glass transition temperature (Tg), such as fiberglass coated with Teflon. This prevents the dry material from sticking to the surface of the film [37,38]. In the last few years, fruits impregnated with probiotics and dehydrated have been studied using hot air drying [11,16,39,40,41,42], vacuum drying [16,21,43], freeze-drying [13,19,44], ultrasound drying [45], and infrared drying [46] technologies. The results of these studies have shown, in general terms, that the survival of probiotic bacteria in dehydrated products is on the order of 10^6^ to 10^7^ (CFU/g), indicating greater survival in the freeze-drying process than in hot air drying and air-drying vacuum; however, freeze-drying requires a long drying time, pretreatment (freezing), high-cost equipment, and energy [47]. The intensified drying characteristics of RW^TM^ may result in the drying of probiotics as long-term heating operations are detrimental to living cells. The RW^TM^ drying of probiotics has been reported for powders [48] encapsulates [49,50], and kefir [51]. Tontul et al. (2018) [52] studied the effect of (RW^TM^) drying in comparison with that of freeze-drying, and the authors reported that the RW^TM^ drying process produced better quality yogurt powder products than the freeze-drying process, except for the color properties, while the survival of probiotics decreased inversely with temperature. Despite the aforementioned advantages of RW^TM^, to the best of our knowledge, the use of RW^TM^ during the fruit probiotic carrier drying has not been reported. The purpose of the present study was to evaluate the use of Refractance Window drying (RW^TM^) to produce dry probiotic-enriched apple slices compared with air-dried (CD) and freeze-dried (FD) processes in terms of probiotic viability, color, texture, and total polyphenols. The study included the effect of the drying process on the survival of bacteria during storage.

## 2. Materials and Methods

### 2.1. Materials

The raw material used was apples (cv. Granny Smith) obtained from a local market in the city of Valparaíso, Chile, stored at 2 °C until use. The apples were washed with tap water and dried. Slices 0.0041 ± 0.0003 m thick and 0.04 ± 0.0001 m in diameter were cut using a cork borer. The samples were obtained only from the parenchymal tissue [53], and each slice had an average weight of 0.0044 ± 0.00028 kg. To avoid enzymatic oxidation during the process, the samples were blanched according to a previously described method by [16] with some modifications by immersing the slices in hot water at 90 °C for 1 min. Subsequently, the samples were placed in a cold-water bath (8 °C) for 1 min.

### 2.2. Impregnation of Probiotic Bacteria

In this work, *Lacticaseibacillus rhamnosus* derived from ATCC^®^ 53103™* was isolated from lyophilized commercial capsules (Microbiologics, MN, USA) and used as a probiotic agent to inoculate apple slices. The *L. rhamnosus* probiotic culture was first cultured in MRS broth (Biokar Diagnostics, Allonne, France) at 37 °C for 48 h. The broth was stored as 50% glycerol stocks at 0.5 McFarland turbidity in sterile cryogenic vials. Glycerol stocks were stored at −80 °C until use. Prior to the experiments, the cultures were activated in MRS broth at 37 °C for 48 h. After incubation, the inoculum solution was prepared by transferring fresh MRS broth into commercial pasteurized apple juice (AFE Company, Maule, Chile). The pH of the juice was adjusted with sodium bicarbonate to pH~5.4. The targeted inoculation level was ~10^9^ log CFU/mL. The apple/impregnation solution ratio was 1:5 (*w*/*v*). The apple slices were soaked in impregnation solution applying a vacuum pressure of 20 kPa according to the previously published method of [43], with modifications, using a DVP-1 vacuum pump (Dosivac, Buenos Aires, Argentina) for 15 min, followed by reestablishing atmospheric pressure by keeping the samples in the impregnation solution for 15 min. At the end of impregnation with the probiotic, the samples had a weight of 0.0041 ± 0.00044 kg.

### 2.3. Drying Process

#### 2.3.1. Conventional Drying (CD)

The apple slicing and drying process was carried out in a convection oven (MEMMERT, model UFB400, Büchenbach, Germany) until the samples reached an a_w_ value of less than 0.4 [54]. The drying temperature was 45 ± 0.5 °C [16] for 330 min (the water activity attained for the dried samples was 0.362 ± 0.021). Drying was performed in triplicate. Likewise, the drying kinetics were evaluated using the loss of water from the samples over time. The sampling period to measure the weight change was 10 min to weigh the samples during drying, and a digital balance with a precision of 0.001 g (model JD400-3, Changzhou, China) was used. Sampling was performed in triplicate.

#### 2.3.2. Refractance Window^TM^ (RW^TM^)

A thermoregulated bath (MEMMERT, model WNB22, Büchenbach, Germany) with distilled water at 45 ± 0.1 °C was used. A 0.1 mm thick plastic film (MYLAR, polyethylene terephthalate) was placed in the thermoregulated bath in which the apple slices were placed. Drying experiments were performed in triplicate. Drying was carried out until the samples reached a water activity lower than 0.4 [54]. The drying time was 210 min (the water activity attained for the dried samples was 0.344 ± 0.021). The sampling period used to measure the weight change was 15 min. As in conventional drying, sampling over time was performed in triplicate.

#### 2.3.3. Freeze-Drying (FD)

Freeze-drying of the apple slices was carried out in an Alpha 2-4 LSCplus freeze-dryer (Martin Christ Gefriertrocknungsanlagen, Osterode, Germany), with an operating temperature of 30 °C and a 0.3 mbar vacuum pressure for 24 h. The samples were frozen at −85 °C in a freezer (Haier ULT Freezer, model DW-86W100J, Qingdao, China) for 24 h before the freeze-drying process. The food was placed on three shelves (with an area of 0.021 m^2^ each), with a total load of 45 slices of apples. It is important to mention that the shelves were controlled by a wireless temperature system, which allowed for monitoring of shelf and food temperature during the drying process. The water activity attained for the dried samples was 0.315 ± 0.052.

For the three drying processes, the dehydrated samples were stored in aluminized Ziploc bags at 4 °C [12,55,56] for 4 weeks for the enumeration of microorganisms expressed as a colony-forming unit in g dry matter (CFU/g_db_), water activity, moisture content (g water/g sample), and total color change (∆E). The analysis was performed weekly.

### 2.4. Plate Count Enumeration of L. rhamnosus

To enumerate *L. rhamnosus* colonies, 10 g of probiotic-impregnated apple was mixed with 90 mL of sterile peptone water, homogenizing the mixture. Ten-fold serial dilutions were prepared from the homogenized samples with peptone water. Appropriate dilutions were inoculated in MRS agar (Biokar Diagnostics, Allonne, France), and petri dishes were incubated at 37 °C for 48 h under aerobic conditions. At the end of the incubation, the colonies of *L. rhamnosus* were counted (CFU/g_db_).

### 2.5. Analytical Determinations: Water Activity and Color Change

The water activity (aw), moisture content, and color of the samples were measured before and after drying. The water activity (aw) was determined using a Rotronic hygrometer (HygroPalm HP23-AW-A, Bassersdorf, Switzerland). Measurements were conducted during drying to specify the moment of the end of the process (aw < 0.4). The moisture content of the samples was determined according to the Society of Official Analytical Chemists by drying 5 g of the apple sample under vacuum conditions in an oven at 70 °C for 24 h until a constant weight was obtained [57]. The initial moisture content of the sample was 0.866 ± 0.002 g water/g sample. The change in color (ΔE) experienced by the apple slices during drying was measured using a CR-300 colorimeter (Konica Minolta, Tokyo, Japan). The type of illuminant was D65, and the degree of the observer was 2°. The result was expressed according to the CIELAB color space: L* indicates luminosity, a* corresponds to the red/green chromatic coordinate, and b* indicates the yellow/blue chromatic coordinate. Measurements were conducted in triplicate, and values are expressed as the average of three sample sites. Equation (1) [58] reports how Δ*E* is determined and represents the change in color between impregnated/fresh and impregnated/dry samples. Pathare et al. [59] indicated that for fresh and processed foods, perceptible differences in color can be classified analytically, considering a Δ*E* > 3.

(1) $${∆E}^{*}=\sqrt{{∆L}^{*2}+{∆a}^{*2}+{∆b}^{*2}}$$

### 2.6. Mechanical Properties

A puncture test measured the mechanical properties of dried apple slices. The measures were performed in a Texturometer (Brookfield, Model CT3, MA, USA) equipped with a 2 mm diameter cylindrical puncture probe (TA-39) controlled by the software TexturePro CT (version 1.6). Each apple slice was carefully placed and held on a special platform while the measuring tip penetrated the slice’s center at a constant speed of 2.0 mm/s. The thickness of the samples was measured in millimeters (mm), maximum force values were recorded in newtons (N), and the crispness was represented by the distance to reach the maximum force in millimeters (mm). The experiments were performed ten times for each treatment.

### 2.7. Determination of Total Polyphenol Content (TPC)

Polyphenols were obtained by extracting two grams of ground sample (fresh, impregnated, or dried) with 20 mL of an 80% methanol solution using a homogenizer at room temperature and protected from light for 1 h. The supernatant was filtered through Whatman paper No. 2 and stored at −20 °C.

The total polyphenol content in the extracts was determined using the Folin–Ciocalteu method [60] and expressed as gallic acid equivalents (GAEs) in mg/g dry matter (mg/g_db_).

The total polyphenol content was determined using a Genesys 5 spectrophotometer (Spectronic Instrument, Inc., Model 336001, Rochester, NY, USA) by measuring the absorbance at 765 nm. The analyses were conducted in triplicate.

### 2.8. Microstructural Analysis

To observe the impregnation of the probiotics in the apple slices, a structural analysis was performed using a Carl Zeiss EVO MA 10 scanning electron microscope (Carl Zeiss SMT, Ltd.,Oberkochen, Germany) at a pressure of 6 × $10^{-6\;}$ mbar and a pressure acceleration voltage of 10 kV. The dried samples by the three methods (CD, RW^TM^, FD) were dewaxed, gold coated, placed on aluminum plates, and secured with double-sided carbon tape.

### 2.9. Effective Moisture Diffusivity (Deff)

The most traditional mathematical model for describing the drying process is the second Fick’s law model [61], which is used for an infinite slab and is represented by Equation (2):

(2) $$MR=\frac{W_{t}-W_{e}}{W_{0}-W_{e}}=\frac{8}{\pi^{2}}\sum_{i=1}^{\infty }\frac{1}{{\left(2i-1\right)}^{2}}e^{\left(\frac{-{\left(2i-1\right)}^{2}\;\pi^{2}\;D_{eff}\;t}{4L^{2}}\right)}$$

where *MR* is the moisture ratio, *W_t_* is the moisture content at any time *t* (g water/g dry basis), *W_e_* is the moisture content at equilibrium (g water/g dry basis), *W*_0_ is the initial moisture content (g water/g_db_), *D_eff_* is the effective diffusion coefficient (m^2^/s), L is the half-thickness of the slice (m), and *t* is the drying time (s). The value of *W_e_* was obtained by checking the asymptotical value of the moisture profile.

The drying process was also analyzed with an anomalous diffusion model based on fractional calculus [62,63,64]. For one dimension, the model is represented by Equation (3) as follows:

(3) $$MR=\frac{W_{t}-W_{e}}{W_{0}-W_{e}}=\frac{8}{\pi^{2}}\sum_{i=1}^{\infty }\frac{1}{{\left(2i-1\right)}^{2}}{E_{\alpha }}^{\left(\frac{-{\left(2i-1\right)}^{2}\;\pi^{2}D_{eff}^{*}\;\;t^{\alpha }}{4L^{2}}\right)}$$

where, D*_eff_ is the moisture effective diffusion coefficient (m^2^/s^*α*^), *t* is the drying time (s), L is the half-thickness of the slice (m) and *E_α_* is the Mittag–Leffler function [25]. The *α* value indicates the transport mechanism that dominates the mass transfer process. Then, if 0 < *α* < 1, the diffusion mechanism can be assumed to be subdiffusive. In contrast, if *α* > 1, the mechanism can be considered to be superdiffusive. If *α* converges to the unit, the anomalous diffusion model converges to Fick’s second law.

*D_eff_* and D*_eff_ were obtained by fitting the MR data with Equations (2) and (3), respectively, by minimizing the sum of the square error using MATLAB R2019a software, version 9.6 (The MathWorks, Inc., Natick, MA, USA).

### 2.10. Statistical Analysis

The significance test of the results was performed with an analysis of variance (ANOVA) and Duncan’s multiple range tests with a significance of 95%. This analysis was conducted using STATGRAPHIC 18 Centurion XVIII software**^®^** (StatPoint Technologies, Inc., Warrenton, VA, USA).

## 3. Results and Discussion

### 3.1. Survival of Probiotics and Microstructure Analysis

The initial inoculation density was 8.53 ± 0.38 log CFU/g_db_, indicating good adaptation and attachment of bacteria on the apple surfaces. Table 1 shows the counts of probiotics on dried apple slices and during storage for 4 weeks at 4 °C. At the beginning of storage, the microbial contents of CD, RW**^TM,^** and FD were 6.30, 6.67, and 7.20 log CFU/g_db_, respectively. The results indicate that the bacterial cells attached to and survived better on the FD-treated apple surfaces than on the CD- and RW^TM^-treated apple surfaces. Similar results have been reported for CD in apple [19,40,65] and murta [43] where impregnation and survival of microorganisms during drying ranged from 10^6^–10^7^ CFU/g. On the other hand, the higher cell concentrations achieved after FD may be related to the mild conditions of this technology compared with the CD and RW^TM^. In these, the heat treatment induces more harmful effects on microorganisms, which can help the viability of the probiotics [52]. On the other hand, Betoret et al. [66] indicated that the inclusion of probiotics in the porous structure of apples through vacuum impregnation could confer protection against cellular damage caused by freezing and subsequent sublimation of frozen water. However, FD is a very slow and expensive drying process: to achieve similar water activity (aw < 0.4), the FD needed 24 h while the CD and RW^TM^ took 5.5 and 3.5 h, respectively.

**Figure 1:**
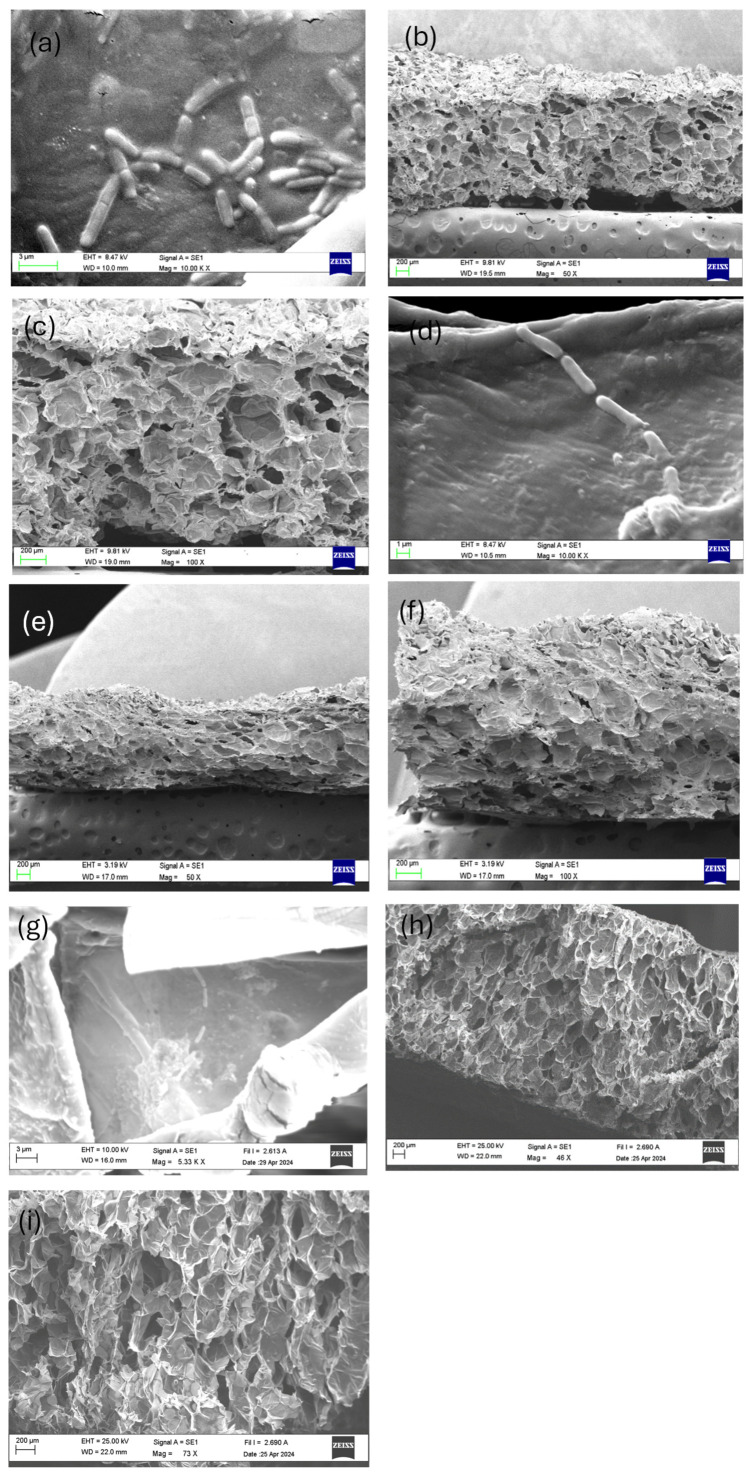
SEM micrographs of the probiotic-enriched apple slices dried using conventional drying (CD) (**a**–**c**), Refractance Window drying (RW^TM^) (**d**–**f**), and freeze-drying (FD) (**g**–**i**) (magnification of 46X (**h**) 50× (**b**,**e**), 73X (**i**), 100X (**f**,**c**), 5330X (**g**) and 10,000X (**a**,**d**), 20 kV).

The physicochemical changes during the drying processes must be considered because they affect the structure of the dehydrated apple matrix [67,68]. Several authors have reported differences in the microstructure caused by different conditions and types of drying [51,69,70,71,72]. Figure 1 shows the micrographs obtained from transverse and superficial sections of dehydrated apple slices using CD (Figure 1a–c), RW^TM^ (Figure 1d–f), and FD (Figure 1g–i) methods. SEM micrographs of probiotic-enriched apple slices were analyzed to observe the surface structure of the dried apples and the presence/attachment of *L. rhamnosus* to the apple surface tissue. The SEM images demonstrate that cells were successfully impregnated onto the apple surface by CD, RW^TM^, and FD drying and not inside the matrix. This difference may be due to the size of the cells. These results show that the apple surface tissues had an adequate structure and sufficient space for the attachment of bacterial cells, which agrees with the findings of [16] and [73]. Figure 1a–c shows that the apple slices dried by CD had a rough surface morphology, while the surfaces of the RW^TM^ samples (Figure 1d–f) were relatively smooth, which could be attributed to the apple slices being in contact with Mylar film during RW^TM^ drying. These results agree with those of [26] and [74] who reported on RW^TM^ drying of berries and mangoes, respectively. Figure 1g–i shows that the apple slices dried by FD had a more porous structure; this phenomenon can be explained by the nature of the FD process, in which the ice in the food materials allows for the structure and shape to be preserved and helps prevent collapse, so there are no significant changes in the volume [28,74].

Another relevant factor is the viability of probiotics during storage and their relationship with the microstructure. Initially, it would be expected that greater porosity and less shrinkage would result in greater stability of the probiotic bacteria in the samples subjected to FD. However, the aw achieved in these samples was considerably lower compared with the other drying methods evaluated. Moreover, for the case of FD, a significant decrease (*p* < 0.05) in the microorganism content was observed with time. Similar results were obtained by Betoret et al. [20] who studied the drying of *L. salivarius* spp. in apples with a storage period of 28 days. In that case, the viability of the probiotics was 79.7% for CD versus 45.1% for FD. This may be explained by the lower moisture content of the FD product. Although *Lactobacilli* sp. can easily adapt to changes in their environment, a lower water content leads to cell membrane contraction with decreased cytoplasmic pH and an increase in the concentration of fruit acids [17]. In addition, Semyonov et al. [75] reported that a significant loss of viability of probiotics can occur during drying and storage due to changes in the physical state of membrane lipids or in the structure of cell protein bacteria caused by high evaporation rates, thus affecting the cell integrity. In this sense, controlled shrinkage may provide an additional barrier of protection to bacteria against to the environmental conditions encountered during drying. This may favor a greater survival in CD and RW compared with FD [76]. On the other hand, the glassy state is an important factor for bacterial stability [28,77]. Tymczyszyn et al. [78] suggested that at high drying rates and rapid diffusion of water occurring in CD samples, no decrease in the probiotic population was observed during storage. These results are different from those reported by [16] since they reported an approximately 1 log reduction in probiotic numbers in apples dried via CD at the same temperature. This difference could be explained by the fact that, in our study, apple juice was used as the impregnation solution, which could have helped maintain the probiotic population during storage. Several factors affect cells physiologically, and pH is one of the most important. Apple fruit had a pH between 3 and 4, and the optimal initial pH value for the growth of *L. rhamnosus* is in the range of 4.5 to 6.4 [79], so the juice that was used (pH~5.4) as a liquid vehicle for microorganism growth, and their incorporation into the apple matrix could have helped to balance the pH. Betoret et al. [76] reported that the use of juice seemed to confer some protection to probiotic cells.

### 3.2. Water Activity and Moisture

Water activity (aw) is an essential parameter in drying because it is an indicator of microbial and physicochemical changes in food products. Table 2 shows the changes in water activity and moisture content of the probiotic-enriched dried apple samples. As shown in this table, on day 0, the water activity and moisture content of the samples were 0.315 to 0.362 and 0.007 to 0.094 g water/g sample, respectively. Then, significant increases (*p* < 0.05) in the aw and moisture content were observed from day 7 to day 14 in the case of RW. The increase in moisture content in dehydrated snacks appears to be related to the gradual migration of moisture into the packaging where an increase in the concentration of reducing sugars is expected. Hydrophilic solutes strongly interact with water, altering the structural associations and mobility of adjacent water. Therefore, the initial interaction between the OH groups of the water in the package headspace and the sugars in the snack could influence the absorption of water. This process is facilitated by the physical structure of the snack (porous and nonhomogeneous), and it has been reported in apple snacks dehydrated by hot air-drying processes [80]. On the other hand, the FD decreased the aw value. The reduction in water content of freeze-dried apple slices during storage could be attributed to the residual moisture present in the food matrix. This trapped water can be released in an easier way than, as in the case of CD, during the storage period because the structure of freeze-dried slices is more open and porous. This fact allows the water molecules to move within the matrix and be released during storage. Similar results were reported by Antal et al. [81] for the case of FD blueberries where the studies showed that the moisture content decreased continuously during the storage period.

### 3.3. Color

The fresh apple slices had L* = 73.09 ± 1.44, a* = −6.48 ± 0.25 and b* = 22.14 ± 0.82, similar to the findings of [17,30]; on the other hand, the impregnated apple slices had L* = 69.92 ± 1.22, a* = −5.69 ± 0.32 and b* = 23.64 ± 1.08, with no differences observed between the impregnated and fresh samples (*p* ≥ 0.05). Therefore, the impregnation solution did not significantly affect the color, as was also shown by [82] with *L. rhamnosus* cultures added to peach jam. The color difference (${∆E}^{*}$) was calculated by comparing the treatments (day 0) with the impregnated sample. The values obtained were 16.09 ± 1.54, 14.51 ± 0.68, and 12.10 ± 1.03 for CD, RW^TM^, and FD, respectively. No significant differences were detected between CD and RW treatments (*p* ≥ 0.05); on the other hand, significant differences were observed compared with those in the FD process, which presented a lower color difference. These results agreed with the results reported by [83,84,85]. In general, it is clear from the literature that color changes in dried products can be affected by many factors related to the processing steps, such as the drying method, and in this sense, many studies have reported that RW^TM^ produces better quality in terms of color than CD drying [36,52,62,86]. This could be related to the longer drying time required for CD drying; in this study, the duration of CD was 2 h longer than that of RW (5.5 and 3.5 h for CD and RW, respectively).

On the other hand, during storage, the difference in color (${∆E}^{*}$) between the samples at the beginning (day 0) and for each week was determined, and the results are shown in Table 2. Significant differences (*p* < 0.05) were detected between the CD treatment and the other drying treatments (FD and RW^TM^), and greater color changes after 28 days of storage were observed in the samples that were dried using CD. Akman et al. [16] did not observe significant differences during storage, which could be explained by the fact that unlike [16], in this study, apple juice was used as an impregnation solution. In this sense, the combination of time and temperature promotes the development of caramelization and Maillard reactions as a consequence of drying. The addition of apple juice as an impregnation medium increases the content of reducing sugars available for the development of non-enzymatic browning during the drying process, which concentrates as dehydration progresses. Furthermore, the autocatalytic nature of these reactions leads to the formation of pigments even during storage [87].

The L* value for CD-treated samples significantly decreased (*p* < 0.05) during storage. This could indicate the browning process, but there was no significant difference (*p* >0.05) between the RW^TM^ and FD samples. An increase in the “*a*” value means a redder chroma, which is also indicative of a browning reaction. In this study, the redness was relatively constant during storage for FD; therefore, there was a decreased loss of green color. However, in the case of CD and RW^TM^, a greater value was observed at the end of storage than at the beginning (*p* < 0.05) because of oxidation. The chroma parameter b behaved similarly to the redness parameter during storage and remained constant in FD samples during the 4 weeks of storage. The color stability could be explained by the fact that FD removes water by sublimation of ice at low temperatures and is carried out under vacuum, which reduces the oxygen content in the environment. Both conditions help prevent additional occurrence of enzymatic browning reactions, resulting in the relative stability of the *L*, *a*, and *b* color parameters [88]. Additionally, the non-enzymatic browning often is a result of the severity of the thermal treatment. In this regard, it is expected that dehydration performed with freeze-drying (FD) results in fewer color changes, followed by Refractance Window (RW^TM^), and finally, convective drying (CD) leads to significant increases in color change (Δ*E*). This is consistent with previous studies on the dehydration of apple chips using various technologies where, generally, CD causes visually noticeable changes in the product’s coloration [69,89].

### 3.4. Textural Properties

The textural properties of a dehydrated product play an important role in its acceptability to the consumer. Textural properties depend mainly on the moisture content of the product and are influenced by the type of drying technology [28]. Table 3 shows the maximum force values, crispness, and thickness of apple slices dried by CD, RW^TM^, and FD. It is observed that CD apple slices obtained the highest hardness and lower values of crispness. This can be attributed to the shrinkage of the sample diameter [13], leading to increased thickness and rubberiness; consequently, more force is required to pass through the sample. In contrast, the RW^TM^ drying resulted in apple slices with lower hardness values than oven drying, aligning with previous research studies [35,36]. This difference can be attributed to a lesser shrinkage, resulting in slices with a reduced thickness and a more rigid and crispy matrix. FD samples exhibited the lowest hardness values but higher crispness. Dadhaneeya et al. [90] reported that freeze-dried samples had the lowest hardness values in comparison to RW^TM^, CD, and vacuum drying. This can be attributed to minimal deformation, with the slices perforating almost immediately upon contact. The FD process maintains the original structure of the sample, having the most significant thickness among the samples.

### 3.5. Total Polyphenol Content

The initial total phenolic content (TPC) of the fresh apples was 11.22 ± 0.47 mg GAE/g_db_, similar to that reported in [30] for Granny Smith apples. The TPC of the CD, RW^TM^, and FD apple samples had average values of 3.85 ± 0.20, 4.64 ± 0.23 and 4.12 ± 0.13 mg GAE/g_db_, respectively. Akman et al. [16] obtained similar results for CD at 45 °C for 6 h; apple slices were impregnated with probiotics, for which a TPC of 3.41 mg GAE/g_db_ was obtained. Significant differences were evident between the treatments. Compared with those of CD, the TPC of apple slices dried with RW^TM^ increased by 20.5% with respect to that of slices dried with CD, which can be attributed to the rapid heating and the significant reduction in drying time of RW^TM^ (3.5 h) compared with that of CD (5.5 h). Rajoriya et al. [89] also found that RW**^TM^**-dried apple slices had a greater TPC than CD-dried apple slices. An increase of 12.6% was observed when comparing RW^TM^ treatment with FD treatment, and similar results were reported for dragon fruit by [90]. These results are similar to those of other studies [26,78,91], which shows that RW^TM^ technology allows for obtaining products in a significantly shorter time with attributes similar to those of FD. This technology is also inexpensive and has a significantly lower power consumption. That is, RW**^TM^** can consume one-third to one-half of the FD power consumption [92]. The drying mechanisms involved in RW^TM^ are conduction, radiation, and convection. At the beginning of drying, the material to be dried has a high humidity, which absorbs electromagnetic radiation. During the drying process, the humidity decreases, which causes the infrared window to gradually close, and the infrared radiation is refracted due to the refractive index of the material; therefore, conduction becomes the predominant mode for heat transfer. Additionally, due to the convective cooling provided by the ambient air flow over the material to be dried, the product temperature is relatively low during RW^TM^ drying, which also helps maintain quality [28].

### 3.6. Effective Moisture Diffusivity (D_eff_)

**Figure 2:**
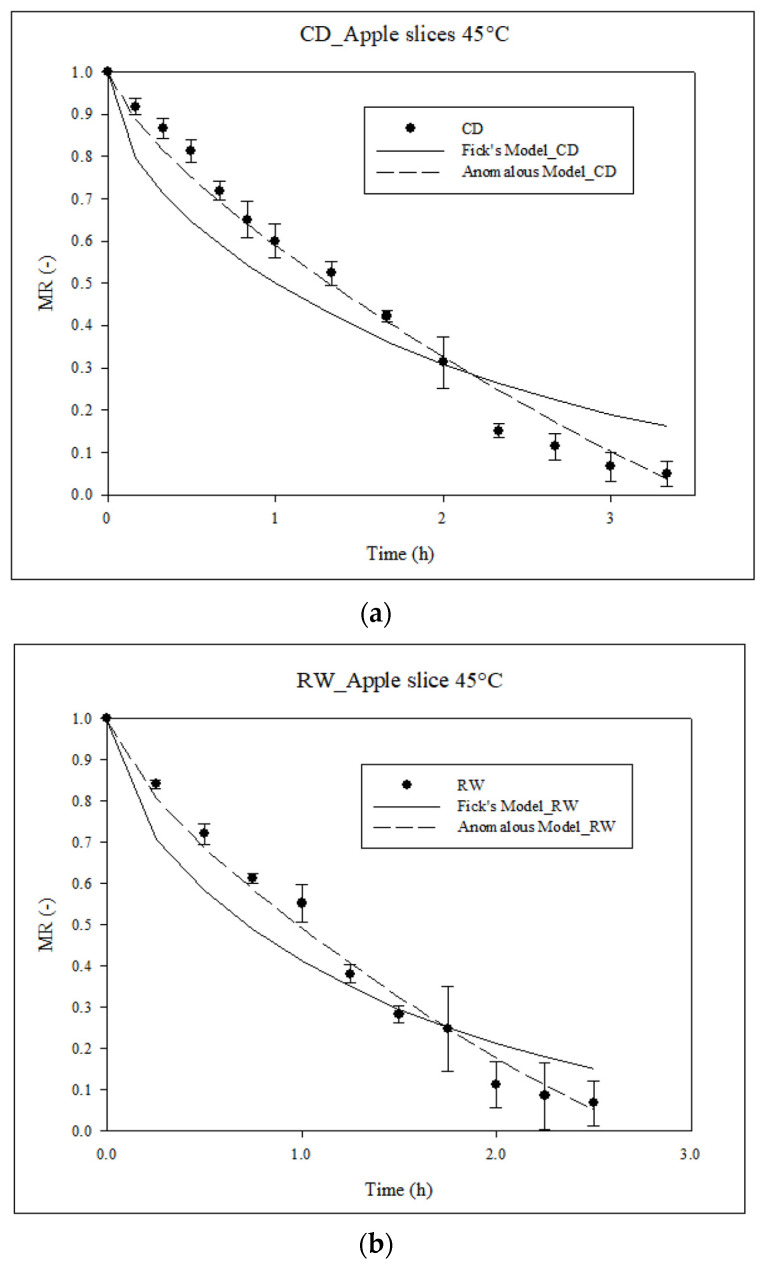
Adjustment of the experimental data of the drying treatments to the Fick and anomalous models: (**a**) SC, (**b**) RW.

The effective diffusion coefficient (*D_eff_*) was obtained by fitting the drying data for apple slices dried using either CD drying or RW™ to Fick’s second and anomalous diffusion models (Figure 2). According to Table 4, the results show that the Fick diffusion coefficient values are between 0.87 and 1.22 × 10^−9^ m^2^/s; on the other hand, the anomalous model coefficient values are between 1.67 and 3.63 × 10^−11^ m^2^/s^α^. It can also be seen that the order of magnitude for *D_eff_* values is lower for the anomalous model than for Fick’s model. According to Rajoriya et al. [31], this difference can be explained by the fact that Fick’s model does not consider a superdiffusive process to calculate *D_eff_*. This indicates that the Fickian approach considers only an exponential type of response. The anomalous model includes the superdiffusive process into *D_eff_* and α terms, which describe a superdiffusion phenomenon (α > 1). This allows us to represent power law kinetics. According to Simpson et al. [25], the time exponent can be related to the microstructure of the food material. The RW**^TM^** values were higher than the corresponding values for CD drying. These results could be explained by the smoother (uniform) microstructure leading to easier and faster diffusion of moisture [31]; on the other hand, in the case of the anomalous diffusion model, it can be observed that in both cases (CD and RW), an α > 1 is obtained, which indicates that the diffusion that occurred during drying in the apple slices was carried out via a superdiffusive process, as suggested by [93,94]. The results showed that the anomalous diffusion model was a better fit (R^2^ > 0.982) than Fick’s model (0.893 < R^2^ < 0.904) for both drying processes. Several studies have concluded that the fit with the anomalous model is better than that with Fick’s model [31,54,62,93]. Vega-Castro et al. [95] concluded that the reason is that the anomalous model has a sound phenomenological description of the process and a high capacity to adjust empirical data.

## 4. Conclusions

Apple slices impregnated with *Lacticaseibacillus rhamnosus* were evaluated and dried using the Refractance Window technology (RW^TM^) to be compared with conventional drying (CD) and freeze-drying (FD). Results indicate that vacuum impregnation with *L. rhamnosus* was successfully achieved, reaching an initial inoculation density of 8.53 log CFU/g_db_. At the end of the drying processes, bacterial viability was best preserved in samples treated by FD, followed by RWTM and CD.

Regarding microstructural changes, the RW^TM^ technology exhibited a smoother surface structure compared with conventional drying, which might facilitate lower mass transfer resistance and more uniform moisture diffusion. These changes are crucial as they directly affect the stability and viability of the incorporated probiotics. At this point, it was found that the effective diffusion coefficient was significantly higher in RW^TM^ drying compared with CD, which can be attributed to the greater uniformity in the microscopic structure observed in the former. The lower drying times of RW^TW^ also yielded samples with a stable delta color during storage and the lowest loss of total polyphenols compared with CD and FD.

Finally, this study provides solid evidence on the effectiveness of the RW^TW^ as a drying technique that not only preserves the viability of probiotics but also maintains structural integrity and nutritional quality of nondairy probiotic foods. These findings contribute to the development of new probiotic products in nontraditional matrices, expanding options for consumers with dietary restrictions or vegan preferences. Future research should focus on optimizing drying parameters, evaluating the long-term stability of probiotics under various storage conditions, and studying in more detail the structural changes occurring in the product during storage and the mechanisms behind them.

## Figures and Tables

**Table 1:** Change in number (log CFU/g_db_) of *L. Rhamnosus* of the apple slices during storage.

	Day 0	Day 7	Day 14	Day 21	Day 28
CD	6.67 ± 0.25 ^a,A^	6.49 ± 0.19 ^a,A^	6.16 ± 0.22 ^a,A^	6.40 ± 0.26 ^a,A^	6.29 ± 0.19 ^a,A^
RW	6.33 ± 0.21 ^a,A^	6.42 ± 0.30 ^a,A^	6.33 ± 0.21 ^a,A^	6.44 ± 0.56 ^a,A^	6.33 ± 0.29 ^a,A^
FD	7.20 ± 0.16 ^b,A^	6.67 ± 0.05 ^a,A^	6.51 ± 0.12 ^a,A^	6.37 ± 0.90 ^a,A^	6.63 ± 0.07 ^a,A^

Different uppercase letters in the same column indicate significant differences (*p* < 0.05). Different lowercase letters on a row indicate significant differences (*p* < 0.05).

**Table 2:** Change of aw, moisture, and color properties of the probiotic-enriched apple slices during the 4-week storage period.

Dehydration Methods	Days	aw	L*	a*	b*	Δ*E*	Moisture (g Water/g Sample)
CD	0	0.362 ± 0.021 ^a^	84.94 ± 0.99 ^a^	−3.63 ± 0.70 ^a,e^	27.98 ± 2.17 ^a^	-	0.084 ± 0.017 ^a,c^
	7	0.439 ± 0.015 ^b^	82.50 ± 1.12 ^b^	−3.92 ± 0.56 ^a,e^	28.10 ± 1.70 ^a^	2.72 ± 0.79 ^a,c^	0.093 ± 0.005 ^a,g^
	14	0.432 ± 0.046 ^c^	82.50 ± 1.93 ^b^	−2.87 ± 0.97 ^b,d^	26.54 ± 2.10 ^a,c^	4.22 ± 2.02 ^a^	0.081 ± 0.008 ^a^
	21	0.455 ± 0.013 ^b^	82.70 ± 2.31 ^b^	−3.07 ± 0.79 ^a,d^	25.73 ± 2.98 ^c^	4.60 ± 1.52 ^a^	0.094 ± 0.002 ^a,h^
	28	0.484 ± 0.028 ^b^	80.32 ± 1.62 ^c^	−2.19 ± 0.64 ^c^	24.87 ± 2.34 ^c,d^	7.81 ± 2.94 ^b^	0.093 ± 0.019 ^a,h^
RW™	0	0.344 ± 0.021 ^a,f^	83.64 ± 0.59 ^a,d^	−1.91 ± 0.40 ^c^	25.71 ± 1.29 ^a,c^	-	0.054 ± 0.006 ^b,d,e^
	7	0.335 ± 0.006 ^a,f,h^	85.03 ± 1.97 ^a,d^	−2.89 ± 0.10 ^d^	21.52 ± 1.14 ^b,e,f,h^	2.65 ± 1.83 ^a,c^	0.058 ± 0.009 ^c,d,e^
	14	0.385 ± 0.004 ^a,c^	83.63 ± 1.40 ^a,d^	−2.87 ± 0.53 ^d^	23.35 ± 1.36 ^f^	3.01 ± 1.86 ^a,c^	0.077 ± 0.012 ^c,d,g,h^
	21	0.393 ± 0.003 ^e,c^	84.17 ± 2.71 ^a,d^	−2.58 ± 0.49 ^d^	23.22 ± 1.19 ^e,h^	3.13 ± 1.18 ^a,c^	0.073 ± 0.007 ^c,d,h^
	28	0.391 ± 0.011 ^c^	84.84 ± 1.37 ^a^	−1.40 ± 0.60 ^c^	19.26 ± 2.37 ^b,g,h^	4.10 ± 1.81 ^a,c^	0.082 ± 0.018 ^c,g,h^
FD	0	0.315 ± 0.052 ^f^	84.67 ± 2.71 ^a,d^	−3.93 ± 0.72 ^e^	20.08 ± 2.51 ^g,h^	-	0.025 ± 0.005 ^e^
	7	0.281 ± 0.052 ^g^	83.83 ± 1.08 ^a,d^	−3.45 ± 0.39 ^e^	19.35 ± 1.29 ^g,h^	1.87 ± 0.79 ^c^	0.023 ± 0.002 ^e^
	14	0.245 ± 0.004 ^g^	84.68 ± 1.27 ^a,d^	−3.34 ± 0.92 ^e^	20.79 ± 2.96 ^g^	2.04 ± 1.02 ^c^	0.015± 0.003 ^f^
	21	0.248 ± 0.006 ^g^	82.89 ± 1.16 ^d^	−3.96 ± 0.35 ^e^	19.89 ± 1.62 ^g^	2.15 ± 1.02 ^c^	0.012 ± 0.006 ^f^
	28	0.286 ± 0.034 ^g,h^	84.20 ± 1.06 ^a,d^	−3.63 ± 0.42 ^e^	18.83 ± 0.84 ^g^	2.29 ± 0.29 ^c^	0.007 ± 0.000 ^f^

Different lowercase letters in the same column indicate significant differences *p* < 0.05.

**Table 3:** Textural properties.

Drying Method	Crispness (mm)	Thickness (mm)	Force (N)
CD	7.46 ± 1.22 ^a^	1.66 ± 0.18 ^a^	27.94 ± 4.37 ^a^
RW ^TM^	4.41 ± 0.76 ^b^	0.77 ± 0.09 ^b^	9.26 ± 0.93 ^b^
FD	2.77 ± 1.31 ^c^	3.70 ± 0.21 ^c^	3.44 ± 0.46 ^c^

a, b, c: Different lowercase letters within the same column and experiment show that the results are statistically significantly different (*p* < 0.05).

**Table 4:** Effective diffusion coefficients for CD and RW drying according to Fick’s second law and anomalous diffusion models.

Drying	Fick’s Second Law		Anomalous Model		
	$D_{eff}(10^{-9}m^{2}/s)$	$R^{2}$	$D_{eff}(10^{-11}m^{2}/s^{\alpha })$	$R^{2}$	α
RW	1.22 ± 0.134 ^a^	0.904	3.63 ± 1.06 ^a^	0.982	1.40 ± 0.02 ^a^
CD	0.87 ± 0.05 ^b^	0.893	1.87 ± 1.25 ^a^	0.983	1.44 ± 0.07 ^a^

Different letters in the same column indicate significant differences (*p* < 0.05).

## Data Availability

The original contributions presented in the study are included in the article, further inquiries can be directed to the corresponding author.
